# Changes of Laboratory Cardiac Markers and Mechanisms of Cardiac Injury in Coronavirus Disease 2019

**DOI:** 10.1155/2020/7413673

**Published:** 2020-05-26

**Authors:** Lin Li, Qi Zhou, Jiancheng Xu

**Affiliations:** ^1^Department of Laboratory Medicine, First Hospital of Jilin University, Changchun 130021, China; ^2^Department of Pediatrics, First Hospital of Jilin University, Changchun 130021, China

## Abstract

Some patients with coronavirus disease 2019 (COVID-19) show abnormal changes in laboratory myocardial injury markers, suggesting that patients with myocardial injury have a higher mortality rate than those without myocardial injury. This article reviews the possible mechanism of myocardial injury in patients with COVID-19. Severe acute respiratory syndrome coronavirus 2 (SARS-CoV-2) affects the patients with COVID-19 in aspects of direct infection of myocardial injury, specific binding to functional receptors on cardiomyocytes, and immune-mediated myocardial injury. During hospitalization, the monitoring of laboratory myocardial injury markers in patients of COVID-19 should be strengthened.

## 1. Introduction

In December 2019, an outbreak of coronavirus disease 2019 (COVID-19) caused by severe acute respiratory syndrome coronavirus 2 (SARS-CoV-2) occurred in Wuhan, China. This has attracted global attention because of its high infectivity. COVID-19 has rapidly spread to more than hundred countries worldwide, of which Western Pacific Region and European Region are severely affected, and there are still new cases appearing. As of May 12, 2020, there were 4,088,848 confirmed cases; of them, 283,153 died [1]. A retrospective review of lung injury caused by COVID-19 found that there were varying degrees of changes in laboratory cardiac markers [2]. Therefore, there is a great deal of attention as to whether it causes myocardial injury. In this paper, we searched for laboratory cardiac markers in patients with COVID-19 to investigate the mechanisms through which SARS-CoV-2 caused myocardial injury.

## 2. Laboratory Cardiac Markers in Patients with COVID-19

The levels of laboratory cardiac markers, lactose dehydrogenase (LDH), creatine kinase (CK), creatinine kinase-muscle/brain activity (CK-MB), myoglobin (Mb), cardiac troponin I (cTnI), alpha-hydroxybutyrate dehydrogenase (*α*-HBDH), aspartate aminotransferase (AST), and N-terminal of the prohormone brain natriuretic peptide (NT-proBNP) increase in different proportions in patients with COVID-19 (see Table 1). Although LDH, CK, *α*-HBDH, and AST are cardiac enzymes, their increases cannot specifically represent myocardial injury. It may be due to damage to the lungs, liver, kidneys, or other organs. However, specific myocardial indicators including CK-MB, cTnI, Mb, and NT-proBNP are increased to varying degrees in patients with COVID-19, especially in ICU and severe patients [3]. A meta-analysis which included 4189 confirmed patients of COVID-19 from 28 studies pointed out that cardiac injury biomarkers rose above normal by the midpoint of hospitalization and spiked immediately before death, which seemed to be the most seen in severe cases [4]. Subsequently, some literatures indicated that increased in troponin I [5], CK-MB, and NT-proBNP [6] were indicators of possible cardiac damaged during SARS-CoV-2 infection. It must be noted that in the clinical scenario of COVID-19 patients, cardiac magnetic resonance or endomyocardial biopsy is rarely feasible; thus, the diagnosis is mainly based on troponin elevation in association with echocardiographic data compatible with acute myocarditis (i.e., segmental wall motion abnormalities, left ventricular ejection fraction (LVEF) < 50%, or the presence of left ventricular wall thickening > 10 mm and/or pericardial effusion) and ECG changes (ST elevation or ST/T segment changes) [7].

Some patients are improved, and laboratory cardiac markers return to normal, whereas some severe patients show further worsening in laboratory cardiac markers, resulting in an irreversible loss. Persistent elevation of laboratory cardiac markers is a prognostic factor of disease worsening [8], and most of these patients are transferred into an intensive care unit (ICU) ward for treatment. There is no evidence that patients with hypertension and cardiovascular and cerebrovascular diseases are more susceptible to infection with SARS-CoV-2, but it is certain that patients with hypertension and cardiovascular and cerebrovascular diseases are more likely to develop severe/ICU cases [9]. On the other hand, patients with SARS-CoV-2 are prone to cardiovascular complications [10]. Li et al. reported that at least 8.0% patients with COVID-19 suffered the acute cardiac injury [9]. The incidence of acute cardiac injury was about 13 folds higher in ICU/severe patients compared with the non-ICU/severe patients [9].

## 3. Mechanism Exploration

### 3.1. Direct Virus Infection

Virus infects cardiomyocytes and replicates intracellularly, resulting in cardiomyocyte degeneration and necrosis, which causes loss of cardiac function and arrhythmia. During this process, the specific binding between viruses and surface receptors in cells is an important event for virus infection of target cells. We investigate the mechanism through which SARS-CoV-2 infects cardiomyocytes to cause direct injury and to analyze receptors that are related to cardiac injury. Hu et al. reported markers of myocardial injury such as troponin T, creatine kinase isoenzyme, and N-terminal of the prohormone brain natriuretic peptide (NT-proBNP) were significantly elevated in patients with nucleic acid test-confirmed COVID-19, who were finally diagnosed coronavirus fulminant myocarditis with cardiogenic shock and pulmonary infection [11]. Subsequently, an otherwise healthy middle-aged white woman with similar symptoms, characteristics, and auxiliary examination results was diagnosed with COVID-19 acute myopericarditis [12]. The two cases suggest that SARS-CoV-2 may directly infect myocardial cells, causing viral myocarditis and impairing myocardial function. However, the above two cases have not undergone pathological examination and cannot confirm the speculation. Recently, endomyocardial biopsy in a 69-year-old patient with acute cardiac injury directly demonstrated low-grade myocardial inflammation and viral particles in the myocardium suggesting either a viraemic phase or, alternatively, infected macrophage migration from the lung, which linked to myocardial localization of SARS-CoV-2 [13]. Subsequent studies had confirmed myocarditis had also been identified with high viral loads and mononuclear infiltration on autopsy of some patients with COVID-19 [14–16]. In fact, one study suggested that up to 7% of COVID-19-related deaths were due to myocarditis [17]. When laboratory myocardial markers elevate and severe arrhythmias appear early in COVID-19, we must be alert to the occurrence of viral cardiomyopathy.

### 3.2. Specific Binding to Functional Receptors on Cardiomyocytes

Angiotensin-converting enzyme 2 (ACE2) is a monocarboxylate that degrades angiotensin II to angiotensin 1–7 [18] and is highly expressed in the lungs and heart [19, 20]. Hofmann et al. proved that ACE2 receptor expression was intimately associated with SARS virus invasion [21], and SARS-CoV infection can lead to infection of ACE2-dependent cardiomyocytes. SARS-CoV-mediated myocarditis was intimately associated with ACE2 [22]. This may be one of the reasons for cardiac insufficiency and poor cardiac outcomes in patients with SARS [22]. The older the person, the poorer the cardiac reserved, which ultimately lead to significant age dependence regarding the SARS mortality rate [23]. Three-dimensional reconstruction and computer simulation experiments that the structure of the receptor binding domain and external region of SARS-CoV-2 is very similar to SARS-CoV, suggesting that ACE2 may be a potential receptor for SARS-CoV-2 and form a tight bond [24]. Letko and Munster successfully proved that SARS-CoV-2 could enter cells expressing human ACE2 [25]. Therefore, it has been fully determined that SARS-CoV-2 enter cells through ACE2 on the surface of human cells [25]. Furthermore, some studies have confirmed that due to the expression of ACE2 cardiomyocytes, SARS-CoV-2 easily invaded cardiomyocytes and caused cardiomyocyte loss [26]. In fact, there are no available data which support that ACE inhibitors (ACEI) increase COVID-19 infection via its binding to ACE2 [27]. However, studies have suggested that although COVID-19 combined with cardiovascular diseases (CVDs) has a higher mortality rate, the use of ACEI/ARB drug intervention has no significant effect on the morbidity and mortality [28]. It is necessary to take ACE2 as the entry point to further study its therapeutic value.

### 3.3. Immune Damage

A paper published in Nature mentioned that the plasma of newly diagnosed patients with COVID-19 contains IL1B, IL1RA, IL7, IL8, IL9, IL10, basic fibroblast growth factor (basic FGF), granulocyte colony-stimulating factor (GCSF), granulocyte-macrophage colony-stimulating factor (GMCSF), IFN-*γ*, IP10, monocyte chemoattractant protein 1 (MCP1), macrophage inflammatory protein 1A (MIP1A), MIP1B, platelet-derived growth factor (PDGF), tumor necrosis factor-*α* (TNF-*α*), and vascular endothelial growth factor (VEGF) [29]. Compared with non-ICU patients, plasma IL-2, IL-7, IL-10, GCSF, IP10, MCP1, MIP1A, and TNF-*α* levels in ICU patients were significantly higher [29]. Subsequent researches have suggested that IL-6, IL-17A, and TNF-*α* are highly expressed in critically ill patients or deaths [30, 31]. Therefore, the blood in the patients with COVID-19, particularly severe cases, contains large number of inflammatory cytokines. The main pathogenesis is characterized by high cell division or “cytokine storm,” resulting in an overexuberant immunologic host response [32, 33]. Virus stimulation will result in the innate immune phase, which is manifested by monocyte and macrophage intervention. As inflammatory mediators, monocytes and macrophages play an important role by activating inflammatory cells to release proinflammatory (stress-activated) cytokines, resulting in “cytokine storm.” A study showed that proinflammatory (stress-activated) cytokines such as TNF-*α*, IL-1, and IL-6 played a role in the pathogenesis of congestive heart failure [34], showing that cytokines played an important role in cardiac injury.

These cytokines act on leukocytes, lymphocytes, platelets, and vascular endothelial cells to secrete inflammatory mediators, which can increase blood C-reactive protein (acute phase protein), *α*2-macroglobulin, and fibrinogen levels while decreasing albumin and transferrin levels [35]. This pathophysiological process causes circulation to be at a high-output and low-resistance state. On the other hand, cytokines such as IL-1, IL-6, and TNF-*α* act on capillaries, resulting in ischemia and hypoxia in peripheral tissues and increased compensatory pulsation to improve peripheral circulation.

When levels of inflammatory cytokines decrease, high output, low resistance, and peripheral circulation are alleviated, and blood circulation returns to normal, thereby alleviating hypotension and tachycardia. A study found that the clinical presentation of SARS-CoV pneumonia was mostly hypotension, tachycardia, impaired systolic, and diastolic functions in the heart [36], which was usually self-limiting [37] and proved the aforementioned speculation. As the disease progresses, the viral load may have returned to normal, but excessive immune responses further damage various major organs, such as the heart. This causes ischemia and hypoxia in cardiac tissues while the heart is overloaded to maintain a high-output and low-resistance state. The further results in ischemic injury and changes in laboratory cardiac markers such as elevated troponin I [5], CK-MB, and NT-proBNP [6].

Li et al. pointed out that due to severe SARS-CoV-2 infection, the pneumonia caused significant gas exchange obstruction, leading to hypoxaemia, which significantly reduced the energy supply by cell metabolism, and increased anaerobic fermentation, causing intracellular acidosis and oxygen-free radicals to destroy the phospholipid layer of cell membrane [9]. Meanwhile, hypoxia-induced influx of calcium ions also led to injury and apoptosis of cardiomyocytes [9]. Generali et al. suggested that a not well-managed inflammatory status led to accelerated atherosclerosis that precipitates ischemic disease; the precipitation of immune complexes on endocardium was finally responsible of inflammatory infiltration which led to subsequent worsening of the previous damage [38]. The blood in the patients with COVID-19, particularly severe cases, contains large number of inflammatory cytokines, suggesting that patients with COVID-19 also experience an overexuberant immunologic host response. It is possible to exacerbate the atherosclerosis that cytokine responses to infection as activators of vascular cells and as inducers of the acute phase response with consequent heightened production of fibrinogen, the precursor of clots, and of endogenous inhibitors of fibrinolysis [39].

Patients with COVID-19 can produce cytokines to enter the systemic circulation, which can stimulate macrophages within the plaque to augment local cytokine production and provoke an increase in tissue factor expression that renders lesions more thrombogenic [40, 41]. If patients with COVID-19 suffer severe underlying atherosclerotic diseases, extreme cases of acute myocardial infarction are likely to occur during the course of the disease. Patients with COVID-19 are likely to suffer atherosclerosis, leading to insufficient coronary blood supply and causing myocardial damage. Interpretation of rises in cardiac troponin requires consideration of the context of the clinical situation. We should be alert to the possibility of other types of cardiogenic diseases [39].

**Figure 1:**
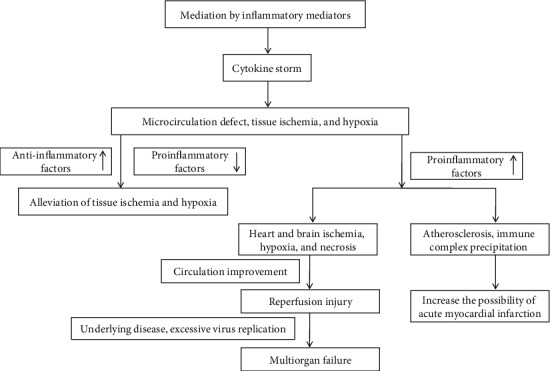
Immune-mediated mechanism of myocardial injury.

The disease is a dynamic process. When ischemia and hypoxia occur in various organs and circulation is not improved, the body will progress to shock. When circulation improves through treatment and ischemic and hypoxic tissues and cells recover, cytokines such as IL-1 [42], IL-6 [43], and TNF-*α* carry out their effects again by participating in ischemia-reperfusion injury and production of large amounts of free radicals, causing tissue damage. This causes secondary damage to organs. Persistent damage to cardiomyocytes causes persistent LDH elevation. A study showed that LDH elevation can reflect the severity of tissue injury and inflammation [44]. Persistent disease progression may lead to irreversible multiorgan failure and can ultimately lead to death (see Figure 1).

## 4. Conclusions

SARS-CoV-2 damages myocardial cells and induces changes of laboratory cardiac markers to varying degrees. The mechanisms include direct infection of myocardial injury, specific binding to functional receptors on cardiomyocytes, and immune-mediated myocardial injury. These mechanisms are not independent and exist strictly in a temporal sequence, as there is a large possibility that these three injury modes simultaneously exist and act together to result in permanent cardiomyocyte loss. Therefore, for patients with COVID-19, it is necessary to actively prevent myocardial injury and reduce the possibility of irreversible remodeling of the myocardium with finally preventing the occurrence of congestive heart failure.

## Figures and Tables

**Table 1:** Cardiac laboratory markers of COVID-19 patients.

Patients	LDH	CK	CK-MB	cTnI	Mb	*α*-HBDH	AST	NT-proBNP
*n* = 41 [29]	29/40 (73) ICU 12/13 (92) No-ICU 17/27 (63)	13/40 (33) ICU 6/13 (46) No-ICU 7/27 (26)	—	5/41 (12) ICU 4/13 (31) No-ICU 1/28 (4)	—	—	15/41 (37) ICU 8/13 (62) No-ICU 7/28 (25)	—
*n* = 99 [45]	75/99 (76)	13/99 (13)	—	—	15/99 (15)	—	35/99 (35)	—
*n* = 188 [2]	129/188 (68.6)	21/188 (11.2)	19/188 (10.1)	21/188 (11.2)	—	143/188 (76.1)	—	—
*n* = 267 [31]	57/267 (21.3) 39/217 (18.0) Nonsevere 39/50 (18.0) Severe	50/267 (18.7) 32/217 (14.7) Nonsevere 18/50 (36.0) Severe	—	3/76 (3.9) 0/55 (0) Nonsevere 3/21 (14.3) Severe	8/76 (10.5) 1/55 (1.8) Nonsevere 7/21 (33.3) Severe	—	—	—
*n* = 140 [46]	—	4/60 (6.7) 1/35 (2.8) Nonsevere 3/25 (12.0) Severe	—	—	—	—	—	—
*n* = 1099 [47]	277/675 (41.0)	90/657 (13.7)	—	—	—	—	168/757 (22.2)	—
*n* = 12 [16]	11/12 (91.7)	1/6 (16.7)	1/12 (8.3)	1/12 (8.3)	2/12 (16.7)	—	2/12 (16.7)	—
*n* = 62 [48]	17/62 (27.4)	5/62 (8.1)	—	—	—	—	10/62 (16.1)	—
*n* = 101 [49]	—	—	32/101 (31.68) Admission to ICU 56/101 (55.45) 48 h to death	51/101 (50.50) Admission to ICU 73/101 (72.28) 48 h to death	—	—	—	37/101 (36.63) Admission to ICU 41/101 (40.59) 48 h to death
*n* = 19 [50]	6/19 (31.58)	1/18 (5.56)	—	—	—	6/8 (75.0)	5/18 (27.78)	—
*n* = 148 [51]	52/148 (35.1)	—	—	—	—	—	32/148 (21.6)	—
*n* = 29 [52]	20/29 (69)	—	—	—	—	—	—	—
*n* = 2 [53]	1/2 (50)	—	—	—	—	—	—	—
*n* = 82 [30]	68/73 (93.2)	25/72 (34.7)	21/70 (30.0)	52/60 (86.7)	42/70 (60.0)	—	44/72 (61.1)	—
*n* = 46 [54]	9/46 (19.6)	2/46 (4.4)	—	—	—	—	3/46 (6.5)	—
*n* = 102 [55]	37/102 (36.3)	19/102 (18.6)	11/102 (10.8)	—	4/59 (6.8)	37/102 (36.3)	26/102 (25.5)	—
*n* = 200 [56]	—	—	—	137/200 (68.5) 112/166 (67.5) Alive cases 25/34 (73.5) Dead cases	—	—	—	—
*n* = 34 [57]	10/34 (29.4)	—	—	—	—	—	—	—
*n* = 9 [58]	—	—	—	—	—	—	3/9 (33.3)	—

Laboratory data is reported as percent of patients with abnormalities defined according to the local reference ranges. No./total No. (%); ICU: intensive care unit; LDH: lactose dehydrogenase; CK: creatine kinase; CK-MB: creatinine kinase-muscle/brain activity; cTnI: cardiac troponin I; Mb: myoglobin; *α*-HBDH: alpha-hydroxybutyrate dehydrogenase; AST: aspartate aminotransferase; NT-proBNP: N-terminal of the prohormone brain natriuretic peptide.
